# Environmental Impact on the Epigenetic Mechanisms Underlying Parkinson’s Disease Pathogenesis: A Narrative Review

**DOI:** 10.3390/brainsci12020175

**Published:** 2022-01-28

**Authors:** Efthalia Angelopoulou, Yam Nath Paudel, Sokratis G. Papageorgiou, Christina Piperi

**Affiliations:** 1 Department of Biological Chemistry, Medical School, National and Kapodistrian University of Athens, 11527 Athens, Greece; angelthal@med.uoa.gr; 2 1st Department of Neurology, Medical School, National and Kapodistrian University of Athens, Eginition University Hospital, 11527 Athens, Greece; sokpapa@med.uoa.gr; 3 Neuropharmacology Research Laboratory, Jeffrey Cheah School of Medicine and Health Sciences, Monash University Malaysia, Bandar Sunway 47500, Malaysia; yam.paudel@monash.edu

**Keywords:** Parkinson disease, epigenetics, DNA methylation, histone modifications, lcRNAs, environmental toxins, smoking, pesticides, coffee, diagnosis

## Abstract

Parkinson’s disease (PD) is the second most common neurodegenerative disorder with an unclear etiology and no disease-modifying treatment to date. PD is considered a multifactorial disease, since both genetic and environmental factors contribute to its pathogenesis, although the molecular mechanisms linking these two key disease modifiers remain obscure. In this context, epigenetic mechanisms that alter gene expression without affecting the DNA sequence through DNA methylation, histone post-transcriptional modifications, and non-coding RNAs may represent the key mediators of the genetic–environmental interactions underlying PD pathogenesis. Environmental exposures may cause chemical alterations in several cellular functions, including gene expression. Emerging evidence has highlighted that smoking, coffee consumption, pesticide exposure, and heavy metals (manganese, arsenic, lead, etc.) may potentially affect the risk of PD development at least partially via epigenetic modifications. Herein, we discuss recent accumulating pre-clinical and clinical evidence of the impact of lifestyle and environmental factors on the epigenetic mechanisms underlying PD development, aiming to shed more light on the pathogenesis and stimulate future research.

## 1. Introduction

Parkinson’s disease (PD) is the most common age-related neurodegenerative movement disorder, affecting approximately 1–2% of the population above the age of 60 years [1]. Neuropathologically, it is mainly characterized by the accumulation of Lewy bodies and Lewy neurites mainly constituting of α-synuclein and the progressive loss of dopaminergic neurons in the substantia nigra pars compacta (SNpc), resulting in nigrostriatal degeneration [2].

Although the exact pathogenic mechanisms remain obscure, several interconnecting pathophysiological processes have been demonstrated to be involved in PD development, including mitochondrial dysfunction, oxidative stress, dysregulation of apoptosis, autophagy impairment, proteosomal dysfunction, and excessive neuroinflammation [2]. Inflammation is rather a “double-edged sword”, since it protects against pathogens and helps to clear out neurotoxins, but it can also induce cytotoxicity and degeneration [3,4]. An inflammatory imbalance, favoring excessive microglial activation (M1 phenotype, pro-inflammatory) against anti-inflammatory responses (M2 phenotype, anti-inflammatory) have been consistently shown to contribute to neurodegenerative disorders, including PD [3,5,6]. Over-activated microglia release pro-inflammatory cytokines, such as TNF-α, IL-6 and IL-1β, induce oxidative stress, α-synuclein accumulation, as well as autophagy and mitochondrial dysfunction, finally leading to a vicious cycle of neurodegeneration [3,7,8]. Viral infections have been also associated with PD progression, potentially acting as triggering factors promoting neuroinflammation [4]. Of note, recent evidence has highlighted the potential role of human endogenous retroviruses (HERVs) in several neurodegenerative diseases [5]. HERVs represent ancient retroviral elements accounting for about 8% of the human genome that can be re-activated by various environmental factors including viruses, resulting in the potential formation of viral particles [5,6]. Although their role in PD pathogenesis has not been elucidated, they could constitute another pathophysiological component of the disease that should be further explored. The peripheral gastrointestinal dysfunction and inflammation have been demonstrated to possibly contribute to dopaminergic neuronal cell death [4]. In addition, the spread and transmission of α-synuclein pathology in a prion-like manner represents another emerging concept in the field of PD pathophysiology that is still under investigation [7]. These diverse pathophysiological contexts highlight the complex but overlapping hypotheses considered to contribute to PD pathogenesis.

Patients with PD display both motor and non-motor manifestations. According to the Movement Disorder Society’s (MDS) PD criteria, parkinsonism is the necessary core feature of PD, defined as bradykinesia plus rigidity or a rest tremor [8]. For the identification of PD as the cause of parkinsonism, there are also absolute exclusion criteria ruling out PD, including cerebellar abnormalities, downward vertical supranuclear gaze palsy, no response to high-dose levodopa treatment, and drug-induced parkinsonism. Red flags for PD diagnosis include rapid progression, early bulbar dysfunction or severe autonomic failure, bilateral symmetric parkinsonism at onset, and early recurrent falls among others. These features should be counterbalanced by additional supportive criteria for PD diagnosis, such as levodopa-induced dyskinesia, rest tremor of a limb, olfactory loss, and a dramatic response to dopaminergic therapy.

Currently, available treatment options mainly involve levodopa and dopamine receptor agonists, which do not halt disease progression, exert only partial symptomatic relief, and are often accompanied by severe adverse effects, such as motor complications (fluctuations and dyskinesias), orthostatic hypotension, and impulse control disorder [2].

Several genetic causes of PD have been identified, including mutations in the *SNCA*, *LRRK2*, *GBA*, *PINK1*, and *Parkin* genes following a Mendelian inheritance pattern, which exhibit variable penetrance and account for only 5–10% of all PD cases [9]. Except for these rare genetic forms, the etiology of most cases of PD remains obscure [10]. Several genes potentially associated with PD risk have also been identified by genome wide association studies (GWAS), including variants of causative genes of PD, such as *MAPT* H1 haplotype, the promoter region of *SNCA* [10], a common polymorphism of *UCHL1*, and a variant of *LRRK2* [11]. Key pathways related to PD susceptibility genes include dopamine transport and metabolism (*DRD2*, *MAO-B, DAT*, and *COMT*), oxidative stress (*SOD2* and *NOS*), and xenobiotics metabolism (*CYP2D6, NAT2*, and *GSTs*) [11,12]. However, many of these studies have provided conflicting results, attributed to ethnic genetic heterogeneities and potential interactions with environmental exposures [11].

Several studies have demonstrated that particular gene polymorphisms interact with exposure to cigarette smoking, pesticides, or coffee to differentially affect the risk for PD development, although with inconsistent results [13]. Advancing age is the strongest risk factor for PD, since its incidence increases exponentially after the age of 60 years [9]. 1-methyl-4-phenyl-1,2,3,6-tetrahydropyridine (MPTP) was first observed to cause parkinsonism in drug users as a contaminant in heroin. MPTP, which is currently widely used as a neurotoxin for mimicking PD in animal models, shares common chemical properties with paraquat, a herbicide [9]. Since then, accumulating epidemiological evidence has shown that other environmental factors, such as tobacco smoking and coffee consumption may protect against PD development, while exposure to pesticides or specific heavy metals including manganese or lead, may increase this risk [14].

However, neither the genetic nor the toxin-based animal models of PD accurately reflect human PD pathology [9]. Therefore, PD is proposed to be a rather multifactorial disorder and a complex interplay between genetic–environmental interactions may underlie its pathogenesis [9]. Pre-clinical evidence has revealed that the cellular effects of several PD-related genes and environmental factors share some common mechanisms, including mitochondrial and autophagy impairment, oxidative stress, and inflammation [9]. Despite extensive research efforts, the exact molecular mechanisms linking these two key disease modifiers are still obscure. In this context, emerging evidence has shown that epigenetics, referring to alterations in gene expression without affecting the DNA sequence, may play an important role in the pathophysiology of neurodegenerative disorders, including PD, potentially representing a mechanistic bridge between sporadic PD and exposure to environmental factors.

In addition, it has been suggested that differences in age of disease onset, severity, and penetrance can possibly be explained by epigenetic modifications [11]. However, till now, very few environmental factors have been demonstrated to cause epigenetic modifications in PD, including smoking, pesticides, and heavy metals [11].

In this narrative review, we discuss the accumulating evidence on the role of environmental and lifestyle factors in epigenetic modifications regulating PD pathogenesis, focusing on smoking, coffee consumption, exposure to pesticides, and specific heavy metals, whose role in PD has been extensively studied. Although the role of epigenetics in PD has already been addressed elsewhere [15,16,17,18], there is no recent review focusing specifically on the epigenetic mechanisms that underlie the pathogenesis of PD from the scope of specific environmental factors. Despite the fact that the field is still in its infancy, we have compiled the available preclinical and clinical results of relative studies, aiming to address the way that exposure to these factors may affect PD development through epigenetic regulation, in order to pave the way for future research on PD pathogenesis, diagnosis, and treatment. Based on the current evidence, potential novel pathways are suggested that need to be explored for better clarification of this interesting relationship.

## 2. Methods

A literature search was performed in the PubMed and Scopus Databases, aiming to identify studies exploring and discussing any concepts on the epigenetic mechanisms underlying the impact of environmental factors on the pathogenesis of PD. We focused on the most well studied environmental factors associated with PD, including tobacco smoking, coffee, pesticides, and heavy metals, and the following keywords were used in various combinations: “epigenetics”, “epigenetic”, “DNA methylation”, “histone modifications”, “histone acetylation”, “miRNA”, “microRNA”, “non-coding RNA”, “lncRNA”, “Parkinson’s disease”, “environmental”, “environment”, “smoking”, “tobacco”, “coffee”, “caffeine”, “pesticides”, “herbicides”, “heavy metals”, “environmental toxin”, “gene polymorphism”, “gene-environment interaction”, and “genetic-environmental interaction”. We searched for both original pre-clinical and clinical studies, as well as reviews, published in the English language, until December of 2021. Although our aim was not to perform a systematic review, we also screened each relevant study for additional results to identify possible further articles that could help to better explore the epigenetic mechanisms mediating the effects of the environmental factors on PD.

## 3. Epigenetic Modifications in PD

**Figure 1:**
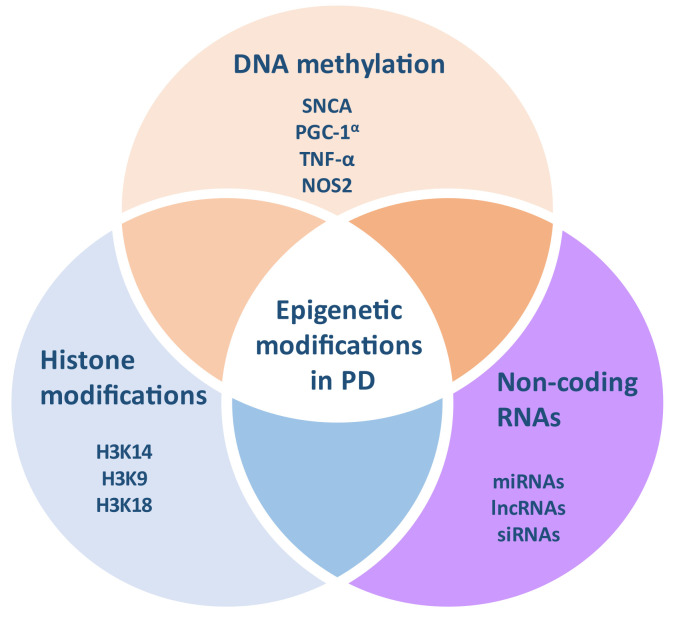
Epigenetic mechanisms regulating the pathogenesis of Parkinson’s disease.

Epigenetic modifications refer to changes in gene expression without affecting the DNA sequence; they occur throughout a lifetime, depending on several environmental factors [10]. Epigenetic mechanisms constitute heritable but possibly reversible alterations, mainly including DNA methylation and post-transcriptional histone modifications, as well as microRNAs (miRNAs) and other non-coding RNAs (Figure 1) [10]. 

Epigenetics are majorly involved in several cellular functions of the central nervous system (CNS), such as neurogenesis, synaptic plasticity, and the learning process. Given the implication of epigenetic mechanisms in both physiological and pathophysiological processes in the CNS [19], it is not surprising that they also play a crucial role in the development of multifactorial disorders, including PD (Table 1).

### 3.1. DNA Methylation in PD

DNA methylation is the most well-studied epigenetic mechanism [39]. The expression levels of many genes depend on the degree of methylation of their promoters [11]. DNA methylation involves the transfer of a methyl group from S-adenosyl methionine (SAM) to the fifth carbon position of the cytosine most common in CpG dinucleotides (CpGs) via DNA methyltransferases (DNMTs), leading to the formation of 5-methylcytosine (5-mC) [40]. Concomitantly, SAM is also converted to S-adenosylhomocysteine (SAH) and finally to homocysteine. CpGs are located at the promoter region of several genes throughout the human genome, known as CpG islands [17]. There are three main types of DNMTs in humans: DNMT1, DNMT3a, and DNMT3b [41]. DNMT1 can bind to hemimethylated DNA, maintaining the patterns of methylation following the replication of DNA, whereas DNMT3a and DNMT3b can bind to both hemimethylated and unmethylated DNA, resulting in de novo methylation [16]. Although the expression of DNMTs is significantly decreased after cellular differentiation, post-mitotic neurons of the mature human brain continue to express DNMTs, suggesting that this epigenetic mechanism could be implicated in neuronal function [40]. DNA methylation may critically affect the interaction between DNA and histones, being functionally associated with gene silencing. This can be mediated directly via the inhibition of the interaction of the DNA machinery with chromatin, or indirectly by recruiting methyl CpG-binding domain (MBD) proteins (MBPs) [17]. On the contrary, unmethylated DNA generally results in gene activation [16]. Thereby, the altered DNA methylation status of gene promoters significantly affects gene expression, contributing to a plethora of pathological conditions.

The ageing process triggers a general reduction in DNA methylation, whereas hypermethylation occurs at certain gene promoters [10]. Disturbed DNA methylation patterns have been identified in the brain and blood of PD patients [42]. DNMT1 has been shown to be downregulated in the brain of PD patients, and reduced DNA methylation was associated with DNMT1 accumulation outside the cell nucleus [20], suggesting that DNMT1 dysregulation may underlie the impaired DNA methylation status in PD. Several genes—including those related to PD development—have been shown to be hyper- or hypomethylated, suggesting that the dysregulation of DNA methylation may be highly implicated in PD pathogenesis [42]. The promoter of the *SNCA* gene was significantly hypomethylated in the brain and blood samples of PD patients [17]. Altered DNA methylation patterns on gene variants of *PARK16/1q32*, *GPNMB/7p15*, and *STX1B*/*16p11* loci in post-mortem brain samples have also been identified between PD patients and controls in a genome-wide association (QWA) meta-analysis [21]. Another study demonstrated that the 5-UTR region of the *dopamine transporter* (*DAT*) gene is hypermethylated in PD patients compared to controls [22]. *DAT* is involved in the maintenance of dopaminergic neurons, and specific *DAT* gene variants have been demonstrated to increase PD risk, although not consistently [11]. Furthermore, the promoter of the *tumor necrosis factor-alpha* (*TNF-α*) gene was found to be significantly hypomethylated in the SN of PD patients compared to controls [23], suggesting that this mechanism could play a role in the excessive neuroinflammation observed in PD. The promoter of the *CYP2E1* gene has also been shown to be hypomethylated in the brain of PD patients [24], and a specific single nucleotide polymorphism (SNP) of this gene has been found to be a genetic risk factor for PD in a Swedish study [43]. Importantly, cytochrome P450 2E1, the protein encoded by *CYP2E1*, is critically involved in the formation of potentially toxic metabolites related to dopaminergic degeneration [16], suggesting that exposure to environmental toxins might contribute to PD pathogenesis via this mechanism. Other genes, including *COMT*—another genetic risk factor for PD—*PRNP*, and *DCTN1* have also shown different DNA methylation levels between PD patients and controls [25].

Collectively, causative genes and gene polymorphisms affecting the risk for PD have been demonstrated to be hypo- or hyper- methylated in PD patients. Some of them are implicated in inflammation and toxin-induced cytotoxicity, two mechanisms critically involved in environmental toxin-induced degeneration [44,45]. Recent evidence has also revealed that specific environmental factors could possibly affect DNA methylation patterns in PD [46]. Thus, our deeper understanding of the exact mechanisms bridging these concepts may open the way for the better elucidation of PD pathophysiology.

### 3.2. Histone Acetylation and Other Modifications in PD

Histone modifications constitute additional epigenetic mechanisms involved in PD. Histones are important nuclear proteins enriched in arginine and lysine residues and allow DNA winding and the formation of nucleosomes [47]. In this way, histones are highly implicated in DNA replication, the regulation of gene expression, and protection against DNA damage [48]. The core five histones H1/H5, H2A, H2B, H3, and H4 [47] are subjected to many types of post-transcriptional modifications at their N-terminal tails, including lysine acetylation and deacetylation, lysine or arginine methylation and demethylation, serine, threonine or tyrosine phosphorylation, SUMOylation, crotonylation, ubiquitination, hydroxylation, and proline isomerization [17,48]. Histone acetyltransferases (HATs) catalyze histone acetylation, thereby opening the chromatin, making the DNA more accessible to transcription factors and subsequently enhancing gene transcription, while histone deacetylases (HDACs) remove the acetyl groups from histones, leading to tighter chromatin packing and the inhibition of gene expression [47]. In this way, HATs and HDACs significantly affect the level of gene expression, as they are crucially implicated in both normal and pathological conditions.

Although histone post-transcriptional modifications have been demonstrated to be majorly involved in the maintenance and differentiation of dopaminergic neurons [49], their role and the factors affecting these molecular processes underlying PD development have not been clarified yet. The acetylation levels of histones H2A, H3, and H4 were shown to be significantly higher in dopaminergic neurons isolated from the midbrain of PD patients compared to controls [27]. In this study, HDACs were also reduced in 1-methyl-4-phenylpyridinium (MPP(+)-treated neuronal cells, and the brain of 1-methyl-4-phenyl-1,2,3,6-tetrahydropyridine (MPTP)-treated mice, implying that environmental toxins could alter histone acetylation levels in PD [27]. A-synuclein accumulation has been associated with H3 hypoacetylation, and HDAC inhibitors may protect against dopaminergic degeneration in pre-clinical studies [28]. Furthermore, sirtuin 2 (SIRT2), a HDAC, is highly implicated in the core pathophysiological mechanisms of PD, such as α-synuclein aggregation, autophagy, oxidative stress, and neuroinflammation, although with conflicting results; there is evidence on the neuroprotective but also detrimental role of SIRT2 in dopaminergic degeneration [29]. Under conditions of oxidative stress, nuclear α-synuclein can bind to the promoter of the *PGC-1α* gene, encoding a protein acting as a mitochondrial transcription factor [30]. This process leads to its hypoacetylation and downregulation of its expression, finally resulting in mitochondrial impairment and neurotoxicity [30]. In summary, emerging evidence highlights the impaired histone acetylation and HDAC levels in PD, which may be associated with oxidative stress, inflammation, and neurotoxin-induced neurodegeneration, suggesting their potential implication in environmental toxin-induced PD-related pathology.

### 3.3. MiRNAs and Other Non-Coding RNAs in PD

MiRNAs (miRs) are small endogenous single-stranded non-coding RNAs consisting of 20–25 nucleotides, which bind to specific RNAs at their 3′-untranslated region (3′UTR), resulting in the negative regulation of gene expression at a post-transcriptional level.

Accumulating evidence has highlighted the role of several miRNAs in PD pathogenesis; a post-mortem study has shown that 125 miRs were differentially expressed in the prefrontal cortex of PD patients compared to controls [31]. MiR-7, miR-203a-3p, and miR-153 are able to bind to and downregulate the expression of the *SNCA* gene [32]. Furthermore, miR-132 has been demonstrated to be downregulated in rat models of PD, accompanied by lower levels of the nuclear receptor related 1 protein (Nurr1), its molecular target [33]. Clinical evidence has shown that miR-133b levels are reduced in the midbrain of PD patients. MiR-133b significantly regulates the maintenance of dopaminergic neurons via its implication in a circuit that involves the paired-like homeodomain transcription factor Pitx3 [34]. Mir-124 has been shown to modulate dopaminergic neuronal loss, mitochondrial function, autophagy, oxidative stress, and neuroinflammation in PD animal models via several signaling pathways [33]. MiR-205 has been found to bind to the 3′ UTR of *LRRK2* and downregulate its expression, while brain samples from the frontal cortex of PD patients displayed increased levels of the LRRK2 protein and low levels of miR-205, supporting its crucial role in *LRRK2* gene suppression [35]. Furthermore, miR-214 levels have been demonstrated to be reduced after the treatment of cells or mice with MPP+ or MPTP, respectively, accompanied by increased α-synuclein levels in dopaminergic cells [36] while resveratrol reversed these effects. Hence, it could be speculated that other environmental toxins could trigger α-synuclein accumulation by altering miR-214 levels. Mir-26 is also upregulated in the SN and CSF of PD patients compared to controls and downregulated in the blood of PD patients [37]. Therefore, miRNAs can mediate at least some of the main pathophysiological processes of PD, such as inflammation, α-synuclein accumulation, mitochondrial impairment, and autophagy dysfunction via a variety of mechanisms. Since neurotoxins, tobacco smoking, and pesticides also affect the levels of miRNAs in several studies [50,51], their role as mediators of the effects of environmental factors on PD risk deserves further elucidation.

Long non-coding RNAs (lncRNAs) are RNA transcripts longer than 200 nucleotides, which play critical roles in neurogenesis and neuroplasticity, and contribute to the pathogenesis of neurodegenerative diseases including PD [38]. A total of 87 lncRNAs have been identified to be differentially expressed in the SN of PD patients, suggesting that they might be actively involved in the pathogenesis of PD [38].

## 4. Environmental Impact on Epigenetic Modifications in PD

Accumulating evidence has indicated the contribution of several environmental factors on epigenetic modifications implicated in the pathogenesis of PD. Among them, the most prominent are smoking, exposure to pesticides and insecticides, coffee consumption, and exposure to heavy metals (Table 2).

### 4.1. Smoking

Many case-control and prospective cohort studies, as well as several meta-analyses have confirmed the inverse relationship between cigarette smoking and PD, reducing the risk by approximately 50% [9,14]. Regarding the underlying molecular mechanisms, it has been shown that nicotine may act neuroprotectively by interacting with nicotinic acetylcholine receptors [80]. Downstream pathways include the cleavage of poly (ADP-ribose) polymerase-1 (PARP-1) and caspase-3 by nicotine [81]. The epigenetic modifications induced by smoking in PD have not been well-studied; however, it has been demonstrated that smoking-induced differentially methylated regions (DMRs) may display diverse distribution patterns in both hypomethylated and hypermethylated regions between smokers and non-smokers [52]. Interestingly, the differentially expressed genes were shown to be implicated in “immunosuppression” pathways, suggesting that smoking-induced epigenetic modifications may involve immune-related mechanisms in PD. For instance, given the differentially methylated promoter of the *TNF-α* gene in PD [23], the role of cigarette smoking in this relationship should be further explored. Additionally, smoking seems to affect histone acetylation and de-acetylation, and affect the expression of miRNAs in non-nerve tissues [82,83,84,85]. Thus, DNA methylation and possibly other epigenetic modifications might mediate the effects of smoking in PD.

To investigate potential gene–environment interactions in terms of epigenetics in PD, it is important to identify shared relative pathways; in particular, we need firstly to explore the common mechanisms between susceptibility genes and epigenetic alterations induced by specific environmental toxins related to PD. In this context, it has been demonstrated that the combination of AA and GA genotypes of rs4680 of the *COMT* gene and non-smoking was associated with a higher risk of PD compared to the combination of AA genotype and a positive smoking history [86]. Given the diverse methylation levels of COMT in PD patients [25], this epigenetic mechanism could potentially underlie the effects of smoking in PD and should be further investigated.

Specific *CYP2D6* gene variants have been shown to interact with cigarette smoking to alter the risk for PD development [87]. However, compared to the extensive metabolizer CYP2D6 genotype, the poor metabolizer has been revealed to reduce the risk of heavy smoking [53]. In addition, DNA hypermethylation of *CYP2D6*, which is observed more commonly in poor metabolizers, is associated with a lower risk of heavy smoking [53]. Therefore, the different methylation status of *CYP2D6* may also be related to smoking behavior, which could in turn alter the PD risk.

Glutathione-S-transferase (GST) plays a major role in the metabolism of both smoke and pesticides, acting as an endogenous antioxidant [88]. An interaction between smoking and specific *GSTP1* polymorphisms has been identified to affect the risk for PD [89,90], although not in all studies [88]. In addition, increased levels of GSTP1 have been observed in the peripheral leucocytes of PD patients upon MPP+ exposure [91], highlighting its implication in the exposure to environmental toxins in PD development. The role of epigenetic modifications of *GSTP1* has not been extensively studied in PD. However, the differential hypermethylation in the *GSTP1* gene has been identified in other neurodegenerative diseases such as Alzheimer’s disease [92]. Another interesting relationship has been identified between *GSTM1* gene polymorphisms (a type of GST) and smoking in regard to PD risk. In particular, a more prominent negative association between smoking and PD has been observed in individuals expressing GSTM1-1 [90], but there is also evidence not confirming this interaction [89]. Thus, we could speculate that an altered *GST* methylation status induced by smoking could at least partially mediate its protective effects in PD, but this hypothesis deserves further study.

Interestingly, a case-control study has indicated that tobacco smoking may be associated with reduced methylation of the promoter region of *long interspersed nucleotide element-1* (*LINE-1*) retrotransposons in blood mononuclear cells only in controls, but not in PD cases [93]. In addition, the inverse relationship between smoking and PD was stronger in low levels of *LINE-1* methylation and became less evident as *LINE-1* methylation levels were elevated [93]. *LINE-1* DNA sequences exist in many repeats in the whole genome, and those carrying an intact promoter can replicate themselves and integrate in other DNA regions, possibly altering gene expression [46]. Importantly, *LINE-1* sequences can be inserted into neural progenitor cells expressing tyrosine hydroxylase (TH), which can be differentiated into dopaminergic neuronal cells, thereby implicating dopaminergic cell survival and differentiation [46]. Collectively, these results suggest that PD patients and controls may respond differently to smoking and display diverse DNA methylation profiles in *LINE-1*, which might affect PD risk. *LINE-1* methylation correlates with genome-wide methylation [93], and DNA hypomethylation has been associated with oxidative stress [94], organic pollutants [95], and heavy metals [96] in some studies. Therefore, it is tempting to propose that tobacco smoking may act protectively against PD especially in the cases of additional environmental exposures, and smoking-induced alterations of LINE-1 methylation status could underlie these effects.

Furthermore, a network-based meta-analysis of four blood microarray studies has demonstrated that the *Polypyrimidine Tract Binding Protein 1* (*PTBP1*) gene, encoding a protein highly implicated in the mRNA translation and stabilization of insulin and previously related to diabetes, was the most significantly downregulated gene of PD patients compared to controls [97]. Longitudinal analyses have demonstrated that the relative abundance of *PTBP1* mRNA significantly decreased over a 3-year follow-up period. Insulin resistance has been associated with PD in several studies, and PTBP1 modulates the expression of glucagon-like peptide 1 (GLP-1) [98], which constitutes a pharmacological target in clinical trials for PD [99]. Interestingly, a study in patients with intracranial aneurysms and controls has demonstrated that long-term tobacco smoking was significantly associated with increased DNA methylation levels in the promoter of the *PTBP1* gene in blood, resulting in a reduction in gene expression [100]. Taken together, it could be speculated that smoking could play a potential role in the dynamic methylation and expression of *PTBP1* gene in PD, and this interesting association should be investigated.

Smoking behavior, including the tendency to start or the ability to quit smoking, has been also hypothesized to affect the relationship between smoking and PD. The *SLC6A3* gene is a *DAT* gene, a variant of which has been also suggested as a genetic risk factor for PD [101]. It was recently demonstrated that the number of tandem repeats and methylation levels of the first intron of *SLC6A3* gene might be related to nicotine dependence and a potentially increased tendency to start smoking and an impaired ability to quit [55]. Another recent study demonstrated that the DNA methylation rates of *DRD2* in peripheral leukocytes were reduced in PD patients [102]. *DRD2* is considered a risk factor for PD, and *ANKK1/DRD2* genetic region variants have been associated with nicotine dependence in males in a Chinese study [56]. Hence, a reduced nicotine dependence associated with altered *DAT* or *DRD2* methylation could reflect at least partially an additional endogenous feature of patients susceptible to PD, suggesting that non-smoking may not represent an absolute “actual cause” of the disease.

**Figure 2:**
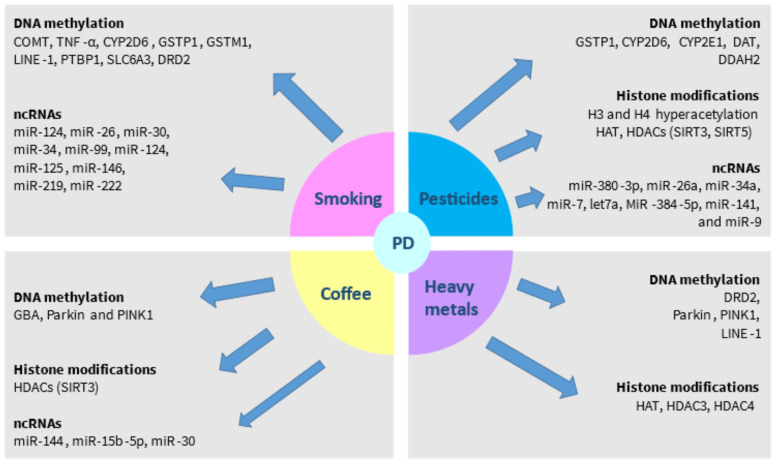
Schematic representation of the potential impact of environmental factors on epigenetic modifications in PD.

Regarding the role of smoking in miRs, a study of the plasma levels of 84 miRs among smokers and non-smokers indicated that miR-124 and let-7a were differentially expressed between these two groups [50]. In addition, nicotine has been demonstrated to attenuate inflammation by upregulating miR-124 [57]. Given the important role of miR-124 in PD as abovementioned [103], the potential effects of smoking on miR-124 levels in PD could also be explored. Moreover, a study that investigated the differential expression of miRs in the lungs of rats exposed to smoking showed that miR-26, miR-30, miR-34, miR-99, miR-124, miR-125, miR-146, miR-219, and miR-222 were among the most significantly downregulated miRs [58]. At the same time, these specific miRs have been associated with PD in various studies [32,37]. These miRs are implicated in stress responses, cell apoptosis, and proliferation [58]. Despite the innate differences between lung and brain tissue, investigating the interaction between smoking and these miRs will enable the elucidation of the molecular mechanisms underlying the protective role of smoking in PD (Figure 2).

### 4.2. Exposure to Pesticides and Insecticides

Several epidemiological studies have demonstrated that exposure to pesticides may increase the risk of PD development [104]. Dieldrin, an organochlorine compound and a persistent organic pollutant widely used as an insecticide, has been associated with an increased PD risk [105]. Preclinical evidence has indicated that dieldrin may enhance reactive oxygen species (ROS) production and oxidative stress, mitochondrial damage, cytochrome c release, and the activation of caspase 3, leading to dopaminergic neuronal apoptosis [106]. Rotenone acts as a neurotoxin by suppressing the mitochondrial complex 1, elevating ROS levels, and inhibiting the production of ATP [107]. Given the effects of pesticides on epigenetic modifications [108], the specific pesticide-induced epigenetic mechanisms in PD have attracted increasing interest.

Several pesticides have been identified as regulators of gene expression at an epigenetic level, including DNA methylation, HDACs, and non-coding RNAs [109]. *Glutathione S-transferase pi* gene (*GSTP1*), which encodes GSTP1-1—a detoxification enzyme—has been shown to increase PD risk with exposure to pesticides [110]. The relationship between the age of PD onset in men and specific *GSTP1* polymorphisms is also affected by the occupational exposure to herbicides [111]. Interestingly, the expression of *GSTP1* is downregulated through the DNA hypermethylation of its promoter by the mutant form *G2019S* of *LRRK2* [112], a genetic cause of PD. In vivo evidence has also shown that the *G2019S LRRK2* mutation was associated with an increased paraquat-induced inflammatory response and an enhanced stress phenotype in transgenic mice [113]. Penetrance of *LRRK2* G2019S is highly variable, estimated at 24–100%, and it is supposed to be affected by ethnicity, gender, and the other genetic or environmental factors modifying the age of disease onset [114]. Therefore, exposure to pesticides may significantly affect the risk and onset age of PD in *G2019S LRRK2* mutation carriers, via the DNA hypermethylation of the promoter of *GSTP1* mediated by the mutant *LRRK2*.

The cytochrome P450 (CYP) genes play a crucial role in xenobiotic metabolism [115]; CYP2D6 activity is majorly affected by common genetic variants in the population, resulting in poor metabolizer phenotypes [87]. Hence, it has been hypothesized that the association between exposure to environmental toxins such as pesticides and PD risk might be affected by CYP2D6 gene variants. In this regard, exposure to pesticides has been demonstrated to potentially modify the effect of CYP2D6 variants on PD risk [116]. It has also been shown that the poor metabolizer genotype of CYP2D6 is associated with a higher DNA methylation status [53]. Hence, impaired DNA methylation might be at least partially responsible for the CYP2D6-dependent effects of pesticide exposure on PD risk.

Furthermore, as abovementioned, the promoter of the *CYP2E1* gene is hypomethylated in the brain of PD patients [24], and its protein product cytochrome P450 2E1 is involved in the formation of toxic metabolites contributing to dopaminergic degeneration [16]. Since paraquat-induced oxidative stress and ROS production is regulated by cytochrome P450 2E1 [117], pesticides might be implicated in PD pathogenesis via altered DNA methylation.

It has been indicated that environmental occupational exposure to the pesticides paraquat and maneb interacted with specific *DAT* gene variants to increase PD risk [118]. Global DNA hypermethylation has been associated with increased concentration of persistent organic pollutants (POPs) in the serum of elder individuals [119]. Given the fact that the *DAT* gene is found hypermethylated in PD patients, this epigenetic mechanism might be at least partially responsible for gene–environment interaction in PD and should be further investigated.

An interaction has been also detected between pesticides and specific SNPs of the *NOS1* gene regarding PD risk in two studies [120,121]. A recent clinical study indicated that the DNA methylation status was different in blood samples from PD patients compared to controls in mitochondria-related genes, including *LARS2*, *MIR1977*, and *DDAH2* [38]. Importantly, DDAH2 modulates the levels of ADMA, which in turn inhibits the activity of NOS [122]. Mitochondrial dysfunction is considered to be a key hallmark of environmental insults [123]. Therefore, a potential interaction between the methylation status of *DDAH2* or other mitochondria-related genes and pesticide exposure should be explored.

H3 and H4 hyperacetylation represents a crucial epigenetic mechanism in dopaminergic neurons upon their exposure to several neurotoxins, including dieldrin, paraquat, rotenone, and MPTP/MPP+ [124]. In particular, dieldrin has been demonstrated to increase H3 and H4 acetylation, potentially leading to proteosomal dysfunction and the accumulation of the cAMP response element-binding protein—aHAT—in mesencephalic dopaminergic neurons [59]. In this study, treatment with the HAT inhibitor anacardic acid prevented against dieldrin-induced histone hyper-acetylation, DNA fragmentation, and dopaminergic degeneration [59]. Similarly, exposure to the herbicide paraquat, which has been also linked with PD, could induce H3 acetylation in dopaminergic cells in vitro, and was also associated with reduced HDAC levels while anacardic acid also protected against these effects [60]. Another study indicated that in paraquat-treated mice α-synuclein was accumulated in the nucleus near acetylated H3, and α-synuclein was able to directly bind to H1 and form a 2:1 complex in vitro [61]. Rotenone has been also shown to promote H3K9 acetylation by downregulating SIRT1 and upregulating p53, thus enhancing neurodegeneration in vitro [62]. Therefore, pesticide induced H3 and H4 hyperacetylation represents a possible mechanism underlying the environmental effects of pesticides in PD, and it could be speculated that HAT inhibitors may inhibit this process.

Variants of *manganese-dependent superoxide dismutase* (*SOD2*), a mitochondrial antioxidant enzyme, have also been associated with an increased risk of PD in some but not all studies [125,126]. Sirtuin 3 (SIRT3), a HDAC, has been shown to deacetylate SOD2, resulting in protection against MPTP-induced ROS accumulation and dopaminergic neurodegeneration in vivo [63]. Another study has indicated that sirtuin 5 (SIRT5), another HDAC, was associated with increased SOD2 levels and improved mitochondrial function in MPTP-treated mice, thereby preventing nigrostriatal degeneration [64]. Therefore, HDACs, including SIRT3 and SIRT5, may act neuroprotectively in neurotoxin-induced PD-related pathology, opening the way for future research on the protection against PD in cases of pesticide exposures.

Several miRNA-related molecular pathways have been implicated in neurotoxin-induced neurodegeneration. For instance, miR-380-3p expression has been shown to be affected by the MPTP–Nrf2 interaction, and the miR-380-3p/Sp3-mRNA pathway was involved in MPTP-induced neurodegeneration [65]. Moreover, rotenone was associated with increased miR-26a and miR-34a levels and reduced miR-7 and let7a levels in rat models of PD [66]. MiR-34a, miR-141, and miR-9 were also differentially expressed in MPP+-treated PC12 cells [67]. MiR-384-5p, which targets and downregulates SIRT1 expression, was increased in rotenone-induced mice and SH-SY5Y cell models of PD [68]. MiR-34a-5p has been shown to play a key role in PD pathophysiology, and differential expression levels have been detected in the plasma of PD patients [69]. Thus, miRNA expression levels seem to be affected by environmental neurotoxins, highlighting their potential role in pesticide-induced PD pathology.

Collectively, this evidence strongly suggests that epigenetic mechanisms including DNA methylation and histone acetylation may underlie the effects of pesticides on PD development (Figure 2). The conflicting results between GWAS may at least be partially explained by the effects of pesticides or other environmental factors on the epigenetic regulation of PD-related susceptibility genes.

### 4.3. Coffee Consumption

PD risk has been shown to be lower in individuals who drink coffee [127,128]. However, there is also evidence that does not confirm this association [129]. Caffeine is considered to act as an adenosine A2A receptor antagonist, inhibiting neuroinflammation and oxidative stress. Recent evidence has also proposed that alterations in gut microbiota are implicated in the relationship between coffee and PD [130]. Concerning the epigenetic modifications underlying the effects of coffee, an association was observed between the DNA methylation status of CpG sites and coffee consumption in some genes causing familial PD, such as *GBA*, *Parkin*, and *PINK1* in the blood of non-PD individuals [70]. Since coffee consumption has recently been shown to possibly protect against early—but not late—onset PD [131], DNA methylation should be further explored especially in these genetic forms of PD.

It has been demonstrated that caffeine may increase the expression of *DAT*, *P450 1A2*, and the *adenosine A2A receptor* in the striatum of MPTP-treated mice [71]. *DAT, P450 1A2*, and *adenosine A2A receptor* gene variants have been already associated with PD risk in some studies, as already mentioned [132]. Although the exact molecular mechanisms remain unclear, epigenetic modifications could possibly underlie these effects, possibly by altering DNA methylation patterns.

Theacrine, a purine alkaloid derived from the Chinese tea “Kucha”, is a chemical analogue to caffeine. A recent study demonstrated that theacrine protected against dopaminergic degeneration in in vitro and in vivo models of PD by directly activating SIRT3, resulting in SOD2 deacetylation, the prevention of apoptosis, the reduction in ROS accumulation, and the restoration of mitochondrial dysfunction [72]. Given the hypothesized similar molecular mechanism of action with caffeine [72], it could be proposed that caffeine might also exert its beneficial effects in PD at least partially through this epigenetic mechanism (Figure 2).

Mir-144 and miR-15b-5p have been shown to be upregulated following treatment with coffee compounds in fibrosis-associated hepatocarcinogenesis mouse models [73]. Furthermore, coffee has been demonstrated to upregulate miR-30 in Caco-2 human colon carcinoma cells in another study [74]. MiR-144, miR-15b-5p, and miR-30 have also been associated with PD pathophysiology [32]. Although innate differences in the gene expression patterns between different tissues should be taken into consideration, the effects of coffee consumption on the regulation of these three miRs should be further investigated as a potential mechanism underlying its effects on PD development.

### 4.4. Exposure to Heavy Metals

Pre-clinical evidence has shown that heavy metals can result in dopaminergic neurodegeneration via several mechanisms including mitochondrial damage, oxidative stress, excessive neuroinflammation, and epigenetic modifications [133]. It has been proposed that increased exposure to heavy metals, such as manganese, copper, mercury, zinc, lead, aluminum, arsenic, and iron increases the risk of PD [134], but the clinical evidence is inconclusive.

A large multicenter study did not indicate any relationship between manganese, iron, and copper and PD risk [135]. On the other hand, manganese in air pollution was demonstrated to be potentially associated with PD development in another Canadian study [136]. Manganese acts as a cofactor for several cellular enzymatic reactions [133], and chronic excessive manganese exposure results in parkinsonian symptoms. Manganese may lead to neurodegeneration by inducing mitochondrial dysfunction, an impairment of energy metabolism, neuroinflammation, and the disruption of synaptic transmission, as well as altering gene expression [137]. A recent in vitro study demonstrated that manganese chloride could inhibit H3 and H4 acetylation, increase HDAC3 and HDAC4 expression, and reduce HAT expression [75]. Furthermore, manganese-treated mice display increased DRD2 expression in their striatum, with unclear molecular mechanisms. Since the DRD2 variant is a susceptibility gene for PD, with PD patients displaying reduced methylation levels of DRD2 [102], it could be hypothesized that manganese could at least partially contribute to PD pathology by modulating *DRD2* gene methylation. Manganese has also been shown to be associated with lower levels of histone acetylation and expression levels of glutamate transporter 1 (GLT-1) and astrocytic glutamate aspartate transporter (GLAST), thereby promoting neurotoxicity [76]. In addition, the gene activities of *Parkin* and *PINK1* have been shown to be affected by increased DNA methylation in vitro upon exposure to manganese [77], suggesting that manganese may also trigger epigenetic modifications in causative PD genes. Thus, manganese may promote dopaminergic degeneration by altering DNA methylation and histone acetylation, although more evidence is needed to clarify this mechanism.

Lead exposure has been also associated with an increased risk for PD [138]. Reduced methylation levels of the promoter of *LINE-1* have been related to exposure to lead [78]. Given the fact that the amount of highly active retrotransposition competent (RC)-LINE-1 has been associated with PD risk and disease progression [139], this epigenetic mechanism could underlie the effects of lead on PD development.

An increased risk of PD has been also associated with exposure to high levels of farm soil arsenic [67]. Although the underlying molecular mechanisms of the effects of arsenic in PD have not been extensively studied, several arsenic-mediated epigenetic alterations have been identified in other conditions, including an impaired *LINE-1* methylation status [79].

## 5. Potential Implications for Diagnosis and Therapy

DNA methylation patterns already represent a promising biomarker for several types of human cancer [140]. Given the implication of impaired DNA methylation in neurodegenerative diseases, it has been proposed that it could also act as a diagnostic or prognostic biomarker for PD [141]. Importantly, DNA methylation alterations in peripheral leucocytes have been well-correlated with those in the brains of PD patients [42], suggesting that blood cells might successfully reflect DNA methylation patterns in the brains of patients with PD and they could be used for this purpose.

DNA methylation rates of *DRD2* in leukocytes were found to be reduced in PD patients but increased in Lewy body dementia (DLB) patients [102]. Given the association between *DRD2* variants and nicotine dependence mentioned before, smoking behavior should be considered as a potential factor contributing to the observed differences in this study.

Importantly, clinical studies have demonstrated that the levels of specific miRs in the blood may significantly differ between PD patients and controls, suggesting that they could be used as potential diagnostic biomarkers. For instance, miR-1, miR-22, and miR-29 were shown to be decreased in non-treated patients with PD compared to controls [142]. Differential expression levels of MiR-34a-5p have been detected in the plasma of PD patients and controls [69], and it has been suggested that it could serve as a potential diagnostic biomarker. However, given the diverse miR-34a levels induced by environmental toxins as mentioned above, exposure to pesticides or other environmental factors should be also considered for the interpretation of these results.

Some of the PD-related susceptibility genes have been also associated with levodopa-induced motor complications in PD patients; for instance, polymorphisms in *MAO-B* and *COMT* genes have been shown to increase the risk of developing dyskinesias and wearing-off [143]. Furthermore, specific *BDNF, DAT*, and *COMT* variants have been demonstrated to exert a synergistic effect on levodopa-induced motor complications [144]. Coffee consumption has been associated with a reduced risk of levodopa-induced dyskinesias in PD patients [145]. Given the potential epigenetic modifications mediated by environmental factors in these genes in PD, a possible effect of these toxins on the development of levodopa-induced motor complications via this mechanism should be further explored.

Pharmaceutical agents acting as epigenetic modulators have been successfully used in cancer. HDAC inhibitors, including vorinostat and agents causing DNA hypomethylation, including azacitidine, have been approved and used against cancer [146]. Novel pharmaceutical approaches targeting epigenetic modifications have also received increasing attention for the treatment of neurodegenerative disorders, including PD. In this regard, DNMT inhibitors exhibited conflicting results in pre-clinical models of PD; it has been indicated that 5-Aza-2′-Deoxycytidine (5-aza-dC), a DNMT inhibitor, can enhance the expression of *TH*—a gene implicated in the production of levodopa—but also the expression of *SNCA* and *UCHL1*, two genes contributing to PD pathogenesis [147].

In regard to potential treatment approaches related to DNA methylation in PD, it has recently been demonstrated that the use of β-naphthoflavone and ethanol, two CYP inducers, in MPP+-treated cells was associated with increased cell viability, lower levels of ROS, the rescue of mitochondrial membrane potential, and the protection of the activity of mitochondrial complex I against MPP+-induced effects [148]. Given the CYP2E1 hypomethylation in PD and its association with the metabolism of pesticides, it could be suggested that these therapeutic agents may prove beneficial in PD-related pesticides exposure and should be investigated. Furthermore, melatonin or silymarin treatment has been associated with the inhibition of paraquat- and maneb-induced dopaminergic degeneration and oxidative damage in PD mouse models, accompanied by decreased CYP2E1 expression [149]. Therefore, the pharmaceutical targeting of CYP2E1 expression via epigenetic modifications represents a promising approach against PD, especially in cases associated with pesticides exposure.

Atremorine, a novel bioproduct from *Vicia faba*, has been shown to act neuroprotectively and enhance dopamine production in PD patients [150]. Interestingly, a recent study revealed that the effects of atremorine as a dopamine enhancer largely depend on variants in several PD-related genes, including *SNCA*, *LRRK2*, *DRD2*, *CYP2D6*, *NAT2, DAT*, and *APOE* among others [150]. The underlying mechanism of atremorine activity is supposed to involve DNA hypermethylation, thereby regulating this extensive pharmaco-epigenetic network [150].

Furthermore, HDAC inhibitors, having been extensively studied in cancer, have also been investigated in neurodegenerative diseases, including PD [17]. Trichostatin A, an agent acting as a HDAC inhibitor leading to increased H3 acetylation, could prevent mitochondrial dysfunction and inhibit neuronal loss in in vitro models of PD, by upregulating (*mitofusin 2*) *MFN2* gene expression [151]. Suberoylanilide hydroxamic acid (SAHA), the first HDAC inhibitor approved for cancer therapy, has been demonstrated to protect against dopaminergic degeneration by enhancing the release of neurotrophic factors from astrocytes [152]. Valproic acid, another widely used anti-epileptic and mood-stabilizing pharmaceutical agent, can also act as a HDAC inhibitor, and promote H3 acetylation; pre-clinical evidence has shown that valproic acid could inhibit neuroinflammation and promote glial cell-derived factor (GDNF) and brain-derived neurotrophic factor (BDNF) expression, resulting in the protection of MPTP- and rotenone-induced dopaminergic neurotoxicity [40]. HDAC inhibitors could be used in case of PD related to pesticide exposure, such as dieldrin and paraquat, since these neurotoxins have been shown to be associated with histone acetylation in PD, as described above.

Moreover, trichostatin A has been demonstrated to protect against manganese-induced neuronal cell death in vitro [75]. Valproic acid could also prevent manganese-mediated decreased histone acetylation and inhibited manganese-induced dopaminergic neurodegeneration [76], highlighting the neuroprotective potential of HDAC inhibitors in manganese-related PD.

However, there is also evidence showing that HDAC inhibitors could be associated with adverse effects in pre-clinical PD models. Sodium butyrate-induced hyperacetylation of histone H4 in mice has been demonstrated to upregulate the protein kinase C δ (PKCδ) in the SN and striatum, enhancing the cellular sensitivity to oxidative stress, potentially resulting in dopaminergic degeneration [153]. Another study indicated that trichostatin A treatment was associated with the reduced survival of dopaminergic neurons [154]. Resveratrol, an agent activating SIRT1—a deacetylase enzyme—has been shown to inhibit rotenone-induced neuronal injury in vitro [62]. Given also the possible diverse responses of HDAC inhibitors in other cell types, further work is needed regarding the clinical effectiveness and safety of these approaches in human PD patients.

Curcumin, a phytochemical with pleiotropic functions, has been shown to protect against PD in several preclinical models [155]. Curcumin acts as a modulator of HATs, HDACs, DNMTs, and specific miRNAs, thereby being involved in several epigenetic modifications [155]. For instance, curcumin displays anti-cancer activities in tobacco smoke-induced lung cancer, by suppressing miR-19 transcription [156]. Hence, it could be speculated that curcumin and possibly other natural products might prevent against PD via epigenetic mechanisms particularly in cases of non-smoking.

Levodopa-induced dyskinesia (LID) represents a late complication of levodopa treatment in PD patients, characterized by involuntary movements often occurring at the peak of dose of levodopa therapy. LID has been associated with the reduced methylation of H3, as well as the deacetylation of histone H4 in the striatum of animal models of LID [157]. Given the protective role of coffee against LID development and PD progression [158], and the neuroprotective effects of caffeine on 6-OHDA-lesioned rat models of PD via histone deacetylase inhibition [159], histone deacetylation could be hypothesized to underlie the protective role of coffee in this case.

## 6. Future Perspectives

Although there are several pre-clinical studies investigating the impact of some environmental factors on epigenetic mechanisms in PD, the clinical evidence is limited. For a better clarification of this process in humans, it would be useful to explore specific epigenetic alterations between PD patients and controls in relation to their exposure to smoking, pesticides, heavy metals, and coffee. In addition, given the impact of environmental factors on the age of onset of the disease in some cases, and the well-known impact of ageing on the epigenome [160], this association requires clarification. Furthermore, given the diverse penetrance of some genetic causes of PD and the interaction between some environmental factors and PD-related genes, the specific impact of environmental exposure on familial cases of PD would be of interest. In this review, some possible mechanistic pathways have been proposed that could be further investigated for this purpose based on evidence from the current literature.

Epigenetic modulators represent an attractive novel approach for PD treatment. In contrast to the irreversible genetic mutations, small molecules targeting HATs, HDACs, DNMTs, miRNAs, and other ncRNAs are receiving increasing research interest. By elucidating the environmental impact on epigenetic alterations in PD, more personalized treatment and preventive approaches could be developed in the future. The deeper understanding of these mechanisms will also allow for earlier intervention at the preclinical stages of PD.

## 7. Conclusions

Collectively, although research on the epigenetic mechanisms underlying the effects of environmental factors on PD pathogenesis is still in its infancy, the combination and analysis of the existing results from relative studies has revealed several possible molecular links between gene–environment interactions that deserve further exploration.

Each PD patient carries a unique combination of various genetic factors that could increase (or decrease) PD susceptibility and is also exposed to a mixture of environmental factors that also affect this risk. Hence, PD is a multifactorial and pathophysiological heterogeneous disorder, in which genetic and environmental factors may rather interact differently in each patient. Further elucidation of the epigenetic mechanisms underlying this interaction will enable an understanding of PD pathogenesis, potentially leading to personalized and more effective treatment approaches.

## Figures and Tables

**Table 1:** Studies revealing the main epigenetic modifications in Parkinson’s disease.

Epigenetic Modifications	Reference
**DNA methylation**	
- DNMT1 is downregulated in the brain of PD patients	[20]
- The *SNCA* gene is hypomethylated in the brain and blood of PD patients	[17]
- Altered DNA methylation patterns on gene variants of PARK16/1q32, GPNMB/7p15, and STX1B/16p11 loci in post-mortem brain samples have also been identified between PD patients and controls	[21]
- The *DAT* gene is hypermethylated in PD patients	[22]
- The *TNF-α* gene is hypomethylated in the SN of PD patients	[23]
- The *CYP2E1* gene is hypomethylated in the brain of PD patients	[24]
- The *COMT*, *PRNP* and *DCTN1* genes are differentially methylated in PD	[25]
- The DNA methylation status is different in blood samples from PD patients compared to controls in mitochondria-related genes, including *LARS2*, *MIR1977*, and *DDAH2*	[26]
**Histone modifications**	
- The acetylation levels of histones H2A, H,3 and H4 are higher in the dopaminergic neurons from the midbrain of PD patients - HDACs are reduced in MPP(+)-treated neuronal cells, and the brain of MPTP-treated mice and	[27]
α-synuclein accumulation is associated with H3 hypoacetylation	[28]
- SIRT2, a HDAC, is implicated in α-synuclein aggregation, autophagy, oxidative stress, and neuroinflammation, although with conflicting results	[29]
- Under oxidative stress, nuclear α-synuclein can bind to the promoter of the *PGC-1α* gene, which leads to its hypoacetylation and the downregulation of its expression, finally resulting in mitochondrial impairment and neurotoxicity	[30]
**Non-coding RNAs**	
- A total of 125 miRs are differentially expressed in the prefrontal cortex of PD patients compared to controls	[31]
- MiR-7, miR-203a-3p, and miR-153 bind to and downregulate the expression of the *SNCA* gene	[32]
- MiR-132 is downregulated in rat models of PD, accompanied by lower levels of Nurr1, its molecular target	[33]
- MiR-133b levels are reduced in the midbrain of PD patients	[34]
- MiR-205 can bind to the 3′ UTR of LRRK2 and downregulate its expression	[35]
- MiR-214 levels are reduced after the treatment of cells or mice with MPP+ or MPTP, respectively, accompanied by increased α-synuclein levels in dopaminergic cells	[36]
- Mir-124 modulates dopaminergic neuronal loss, mitochondrial function, autophagy, oxidative stress, and neuroinflammation in PD animal models via several signaling pathways	[33]
- Mir-26 is upregulated in the SN and CSF of PD patients and downregulated in the blood of PD patients	[37]
- MiR-30, miR-34, miR-99, miR-124, miR-125, miR-146, miR-219, and miR-222 are differentially expressed in PD	[32]
- MiR-144 and miR-15b-5p are associated with PD	[32]
- A total of 87 lncRNAs are differentially expressed in the SN of PD patients	[38]

**Table 2:** Studies of the environmental impact on the epigenetic modifications implicated in Parkinson’s disease.

Environmental Factors	Reference
**Tobacco smoking**	
- Smoking-induced DMRs may display diverse distribution patterns in both hypomethylated and hypermethylated regions between smokers and non-smokers - The differentially expressed genes are implicated in “immunosuppression” pathways	[52]
- The DNA hypermethylation of *CYP2D6*, which is observed more commonly in poor metabolizers, is associated with a lower risk of heavy smoking	[53]
- Tobacco smoking is associated with the reduced methylation of the promoter region of *LINE-1* retrotransposons in the blood mononuclear cells only in controls, but not in the PD cases - The inverse relationship between smoking and PD is stronger in low levels of *LINE-1* methylation and is less evident as *LINE-1* methylation levels are increased	[54]
- Methylation levels of the first intron of *SLC6A3* gene are potentially related to nicotine dependence, an increased tendency to start smoking, and an impaired ability to quit	[55]
*- ANKK1/DRD2* genetic region variants are associated with nicotine dependence in males	[56]
- MiR-124 and let-7a are differentially expressed between smokers and non-smokers	[50]
- Nicotine attenuates inflammation by upregulating miR-124	[57]
- MiR-26, miR-30, miR-34, miR-99, miR-124, miR-125, miR-146, miR-219, and miR-222 are among the most significantly downregulated miRs in the lungs of rats exposed to smoking	[58]
**Pesticides exposure**	
- Dieldrin increases H3 and H4 acetylation, leading to proteosomal dysfunction and the accumulation of the cAMP response element-binding protein in dopaminergic neurons - Treatment with the HAT inhibitor anacardic acid prevents against dieldrin-induced histone hyper-acetylation, DNA fragmentation, and dopaminergic degeneration	[59]
- Exposure to the herbicide paraquat induces H3 acetylation in dopaminergic cells, and is associated with reduced HDAC levels - Anacardic acid protects against these effects	[60]
- In paraquat-treated mice, α-synuclein is accumulated in the nucleus near acetylated H3, and α-synuclein can directly bind to H1 and form a 2:1 complex	[61]
- Rotenone promotes H3K9 acetylation by downregulating SIRT1 and upregulating p53, thus promoting neurodegeneration	[62]
- SIRT3, a HDAC, can deacetylate SOD2, resulting in protection against MPTP-induced ROS accumulation and dopaminergic neurodegeneration	[63]
- SIRT5, another HDAC, is associated with increased SOD2 levels and improved mitochondrial function in MPTP-treated mice, thereby preventing nigrostriatal degeneration	[64]
- The miR-380-3p/Sp3-mRNA pathway is involved in MPTP-induced neurodegeneration	[65]
- Rotenone is associated with increased miR-26a and miR-34a levels and reduced miR-7 and let7a levels in rat models of PD	[66]
- MiR-34a, miR-141, and miR-9 are differentially expressed in MPP+-treated PC12 cells	[67]
- MiR-384-5p, which targets and downregulates SIRT1 expression, is increased in rotenone-induced mice and SH-SY5Y cell models of PD	[68]
- Differential expression levels of MiR-34a-5p are detected in the plasma of PD patients	[69]
**Coffee consumption**	
- Association between the DNA methylation status of CpG sites and coffee consumption in some genes causing familial PD, such as *GBA*, *Parkin*, and *PINK1* in the blood of non-PD individuals	[70]
- Caffeine may increase the expression of DAT, P450 1A2, and the adenosine A2A receptor in the striatum of MPTP-treated mice	[71]
- Theacrine protects against dopaminergic degeneration in in vitro and in vivo models of PD by directly activating SIRT3, resulting in SOD2 deacetylation, the prevention of apoptosis, a reduction in ROS accumulation, and the restoration of mitochondrial dysfunction	[72]
- Mir-144 and miR-15b-5p are upregulated following treatment with coffee compounds	[73]
- Coffee has been demonstrated to upregulate miR-30	[74]
**Exposure to heavy metals**	
- Manganese chloride can inhibit H3 and H4 acetylation, increase HDAC3 and HDAC4 expression, and reduce HAT expression	[75]
- Manganese is associated with lower levels of histone acetylation and expression levels of GLT-1 and astrocytic GLAST, thereby promoting neurotoxicity	[76]
- *Parkin* and *PINK1* gene activities are affected by increased DNA methylation in vitro upon exposure to manganese	[77]
- Reduced methylation levels of the promoter of *LINE-1* are related to exposure to lead	[78]
- Arsenic alters the status of *LINE-1* methylation	[79]

## Abbreviations

PD	Parkinson’s disease
SNpc	Substantia nigra pars compacta
GWAS	Genome wide association studies
MPTP	1-methyl-4-phenyl-1,2,3,6-tetrahydropyridine
miRNAs	microRNAs
CNS	Central nervous system
DNMTs	DNA methyltransferases
MBD	Methyl CpG-binding domain
CpGs	CpG dinucleotides
DAT	Dopamine transporter
HATs	Histone acetyltransferases
HDACs	Histone deacetylases
Nurr1	Nuclear receptor related 1 protein
lncRNAs	Long non-coding RNAs
DMRs	Differentially methylated regions
LINE-1	Long interspersed nucleotide element 1
PTBP1	Polypyrimidine Tract Binding Protein 1
GLP-1	Glucagon-like peptide 1
CYP	Cytochrome P450
